# Extradural Motor Cortex Stimulation in Parkinson’s Disease: Long-Term Clinical Outcome

**DOI:** 10.3390/brainsci11040416

**Published:** 2021-03-26

**Authors:** Carla Piano, Francesco Bove, Delia Mulas, Enrico Di Stasio, Alfonso Fasano, Anna Rita Bentivoglio, Antonio Daniele, Beatrice Cioni, Paolo Calabresi, Tommaso Tufo

**Affiliations:** 1 Neurology Unit, Fondazione Policlinico Universitario A. Gemelli IRCCS, 00168 Rome, Italy; carla.piano@policlinicogemelli.it (C.P.); annarita.bentivoglio@policlinicogemelli.it (A.R.B.); antonio.daniele@unicatt.it (A.D.); paolo.calabresi@policlinicogemelli.it (P.C.); 2 Department of Neurosciences, Università Cattolica del Sacro Cuore, 00168 Rome, Italy; 3 Institute of Neurology, Mater Olbia Hospital, 07026 Olbia, Italy; delia.mulas@gmail.com; 4 Institute of Biochemistry and Clinical Biochemistry, Università Cattolica del Sacro Cuore, 00168 Rome, Italy; enrico.distasio@unicatt.it; 5 Cheminstry, Biocheminstry and Clinical Molecular Biology, Fondazione Policlinico Universitario A. Gemelli IRCCS, 00168 Rome, Italy; 6 Edmond J. Safra Program in Parkinson’s Disease, Morton and Gloria Shulman Movement Disorders Clinic, Toronto Western Hospital and Division of Neurology, University of Toronto, Toronto, ON M5T 2S8, Canada; alfonso.fasano@uhn.ca; 7 Krembil Brain Institute, Toronto, ON M5T 1M8, Canada; 8 Center for Advancing Neurotechnological Innovation to Application (CRANIA), Toronto, ON M5G 2A2, Canada; 9 Neurosurgery Unit, Fondazione Policlinico Universitario A. Gemelli IRCCS, 00168 Rome, Italy; beatrice.cioni@policlinicogemelli.it (B.C.); tommaso.tufo@policlinicogemelli.it (T.T.)

**Keywords:** motor cortex stimulation, Parkinson’s disease, movement disorders, neuromodulation

## Abstract

Previous investigations have reported on the motor benefits and safety of chronic extradural motor cortex stimulation (EMCS) for patients with Parkinson’s disease (PD), but studies addressing the long-term clinical outcome are still lacking. In this study, nine consecutive PD patients who underwent EMCS were prospectively recruited, with a mean follow-up time of 5.1 ± 2.5 years. As compared to the preoperatory baseline, the Unified Parkinson’s Disease Rating Scale (UPDRS)-III in the off-medication condition significantly decreased by 13.8% at 12 months, 16.1% at 18 months, 18.4% at 24 months, 21% at 36 months, 15.6% at 60 months, and 8.6% at 72 months. The UPDRS-IV decreased by 30.8% at 12 months, 22.1% at 24 months, 25% at 60 months, and 36.5% at 72 months. Dopaminergic therapy showed a progressive reduction, significant at 60 months (11.8%). Quality of life improved by 18.0% at 12 months, and 22.4% at 60 months. No surgical complication, cognitive or behavioral change occurred. The only adverse event reported was an infection of the implantable pulse generator pocket. Even in the long-term follow-up, EMCS was shown to be a safe and effective treatment option in PD patients, resulting in improvements in motor symptoms and quality of life, and reductions in motor complications and dopaminergic therapy.

## 1. Introduction

Chronic motor cortex stimulation by implanted extradural electrodes (EMCS) is a minimally invasive therapy proposed as an alternative surgical treatment for Parkinson’s disease (PD) patients who are not eligible for deep brain stimulation (DBS) [1,2]. The pathophysiological rationale for EMCS in PD derives from several clinical and experimental observations. The primary motor cortex (M1) and lateral premotor cortex are hyperactive in advanced parkinsonism, with increased excitability of corticospinal projections at rest, concomitant with or resulting from reduced intracortical inhibition (ICI) [3]. EMCS may restore normal ICI by acting on small inhibitory interneurons within M1 [4]. Indeed, it may act by desynchronizing pathological oscillations in the beta-band between the basal ganglia and cortical neurons, influencing the electrical activity of subcortical structures in primate models [5]. Moreover, functional neuroimaging studies suggest that EMCS might restore the activity of cortical areas that are hypoactive in PD (e.g., the supplementary motor area, SMA) [6].

Initial reports suggesting effectiveness of EMCS in treatment of PD motor symptoms date back almost 20 years [7]. To date, over 100 PD patients have been treated by EMCS, although the available studies concern small samples with a short-term follow-up [2,8,9,10,11,12,13]. Differences among various centers in terms of patient selection criteria, electrode placement and stimulation parameters may explain the inconsistent findings reported so far [13]. Although several Authors have reported that EMCS is safe and may improve PD motor symptoms, studies addressing the long-term outcomes are still lacking [14,15]. Recently, for selected patients, spinal cord stimulation has emerged as an alternative neuromodulation procedure to DBS, emphasizing the need for less-invasive surgical options for PD and effective treatments for axial symptoms [16].

This prospective, single center, open-label study was aimed at assessing the efficacy and safety of EMCS in PD patients with long-term follow-up, up to eight years after surgery. In particular, the following outcomes were investigated: efficacy of EMCS on PD motor symptoms and motor complications (motor fluctuations and dyskinesias); reduction in dopaminergic therapy after surgery; impact of EMCS on daily living activities (DLA) and quality of life (QoL); cognitive and behavioral safety of the stimulation, peri- or post-operative adverse events (AEs).

## 2. Materials and Methods

Consecutive PD patients who underwent EMCS implantation at Fondazione Policlinico Universitario Agostino Gemelli IRCCS in Rome between 2003 and 2007 were included after local ethics committee approval (#400-A763). All patients signed a detailed informed consent form.

All enrolled patients had completed at least a 24-month follow-up and fulfilled the following inclusion criteria: PD diagnosis according to United Kingdom PD Brain Bank criteria [17]; disease duration longer than 5 years; dopaminergic responsiveness confirmed by a pharmacological test showing at least a 33% decrease in the Unified Parkinson’s Disease Rating Scale (UPDRS) motor score [18]; unsatisfactory pharmacological management of fluctuations; lack of eligibility for DBS (i.e., not accepted by patients or contraindicated according to Core Assessment Program for Surgical Interventional Therapies in PD [19]); ability to give informed consent; stable drug regimen and motor condition for at least 3 months preoperatively.

Exclusion criteria included: history of epilepsy or epileptic activity on electroencephalography; alcohol or drug abuse; previous brain surgery; severe psychiatric symptoms (e.g., psychosis, major depression); moderate or severe cognitive impairment (score < 24 on the Mini-Mental State Examination [20]); diagnosis of dementia according to the Diagnostic and Statistical Manual of Mental Disorders, Fourth Edition (DSM-IV) [21]; medical condition contraindicating a surgical procedure under general anesthesia.

### 2.1. Surgical Technique

**Figure 1:**
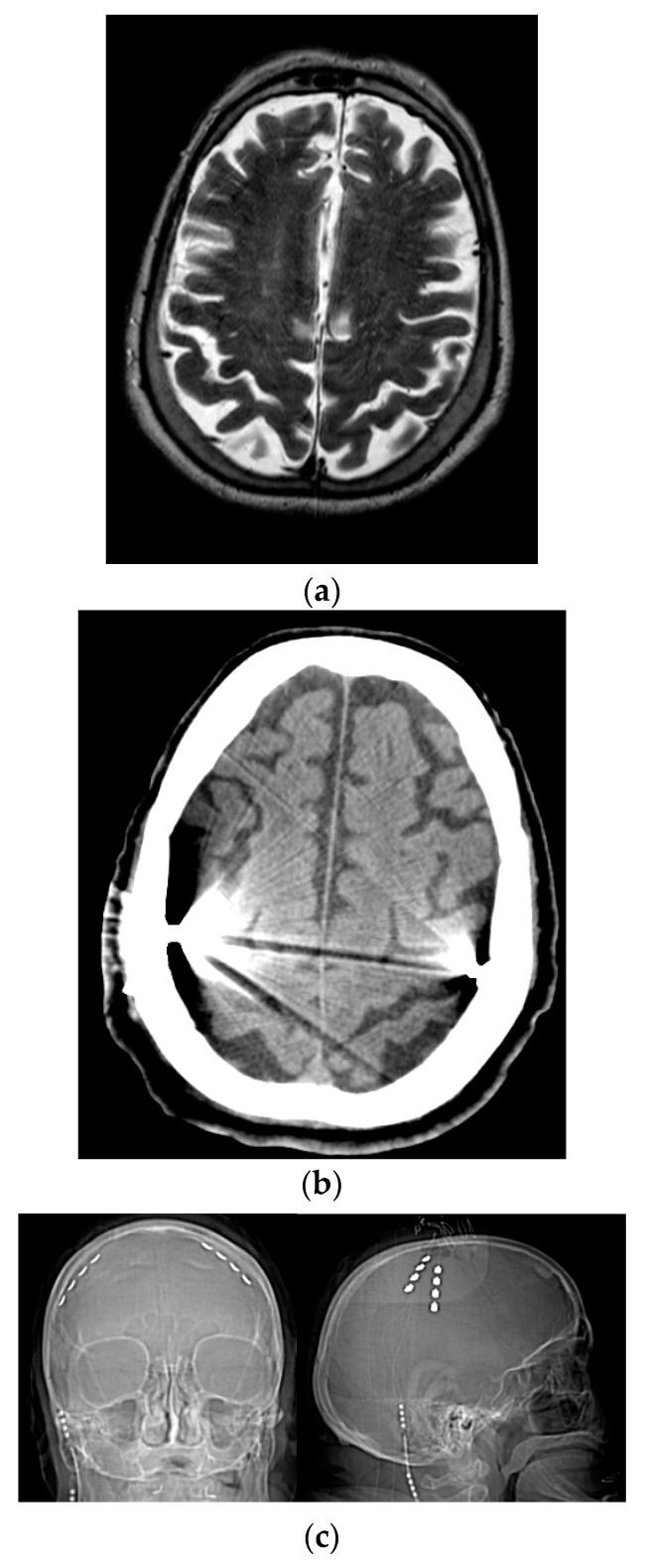
Placement of quadripolar electrode strips over the motor cortices. (**a**) Preoperative brain MRI. (**b**) Postoperative brain CT scan. (**c**) Postoperative skull X-ray. R = right; L = left.

The surgical procedure was performed with patients under total intravenous anesthesia: a quadripolar electrode strip (model Resume; Medtronic Inc, Minneapolis, Minnesota) was epidurally placed over M1 (through a burr hole over, contralateral to the most affected body side in three patients and bilaterally in remaining patients) and connected to a Soletra or Kinetra (Medtronic Inc) implantable pulse generator (IPG) located in the subclavian region. In all patients, contacts were oriented along the craniocaudal axis of the precentral gyrus: contact 3 was 2 to 3 cm from midline, contact 0 was 4 cm more lateral (Figure 1). Implantation site was preoperatively defined (using magnetic resonance imaging and neuronavigation) and verified by means of motor-evoked potentials and by identifying N20-P20 phase reversal of somatosensory evoked potentials obtained from contralateral median nerve stimulation [22]. Patients postoperatively underwent a computed tomography scan to confirm that the electrode paddle was correctly placed and to rule out surgical complications.

### 2.2. Parameter and Medication Adjustments

Parameter setting was performed in the weeks following surgery. In all patients, stimulation was unilateral (namely, contralateral to the body side with more severe motor impairment) for the first 12 months, and afterwards was bilateral for six patients with electrodes over both hemispheres. Stimulation was continuously delivered through the two most distant contacts of the electrode paddle under the bipolar setting. Stimulation parameters were: biphasic wave of 120 μs duration and 80 Hz frequency; voltage maintained at 50% of threshold intensity for any movement or sensation (between 3 and 5 V). EMCS parameters were stable during the first 24 months of follow-up, to evaluate the chronic effects of stimulation with constant parameters. Additional parameter adjustments (voltage increasing or reduction) were attempted according to clinical response and electrode impedances, after 24-month follow-up. Antiparkinsonian medications were gradually decreased according to individual clinical condition.

### 2.3. Outcome Measures

Patients were evaluated at preoperative baseline and postoperatively (12, 18, 24, 36, 48, 60, 72, 84 and 96 months after implantation). Evaluations were performed in the morning, in the practically defined off-medication condition (off-med, at least 12 h after medication withdrawal) and in the on-med condition, following administration of a standard liquid levodopa at doses 50% higher than the usual morning dose of dopaminergic treatment [19]. Postoperative assessments were performed during the on-stimulation condition since a carryover effect was expected to last several days after switching off pulse generator [6]. Motor evaluation was video-recorded for independent analysis. Outcome measures included the UPDRS subscales I, II, III (in off- and on-med), and IV [18]. Arising from chair, posture, gait, postural stability, body bradykinesia (items 27–31 of UPDRS-III) were also separately scored and a total axial subscore (range 0–20) was calculated by addition of these scores. Disease severity was measured by the UPDRS total score (i.e., the sum of UPDRS I, II, III (only in off-med condition) and IV). At each evaluation, QoL, by means of the PD QoL questionnaire (PDQL) [23], and levodopa equivalent daily dose (LEDD) [24] were assessed.

Cognitive and behavioral assessments were performed at preoperative baseline and postoperatively (at 12, 18, 36, 60 and 96 months after implantation). Cognitive assessment was performed by means of a neuropsychological test battery [25], including Mini-Mental State Examination (MMSE), tasks exploring visuospatial (Corsi Block-Tapping Test) and verbal (digit span forward and backward) working memory, episodic verbal memory (Rey’s Auditory Verbal Learning Test, RAVLT), nonverbal abstract reasoning (Raven’s Progressive Matrices ’47, RPM ’47), phonological and semantic verbal fluency, problem-solving and set-shifting abilities (modified Wisconsin Card Sorting Test, mWCST), response inhibition (Stroop test). Tests sensitive to motor speed were not included in neuropsychological tasks, to minimize the bias due to motor impairment. Behavioral assessments included Zung’s Self-Rating Depression and Anxiety Scales [26,27], and a clinical interview aimed at detecting behavioral abnormalities or psychiatric disorders. Cognitive and behavioral assessments were performed in the on-med condition.

Stimulation- or device-related AEs were collected at each postoperative evaluation. An electroencephalogram was recorded preoperatively, and postoperatively at 6 and 12 months.

### 2.4. Statistical Analysis

Data were analyzed for normality of distribution using the Kolmogorov–Smirnov test of normality. Continuous data (comparisons between preoperative and postoperative scores at each follow-up visit up to 72 months) were analyzed by means of the Wilcoxon signed-rank test, and are presented as mean ± standard deviation. Comparisons between categorical variables were performed by Fisher’s test. Given the explorative nature of our study, the standard non-corrected significance α level of *p* < 0.05 was used to reduce the risk of a type II error. However, considering the risk of a type I error deriving from multiple comparisons, significant values should be interpreted with cautions when levels are only marginally lower than 0.05. All statistical computations were two-sided and relied on Statistical Package for the Social Sciences (SPSS) software, version 15.0 (IBM Co., Armonk, NY, USA).

## 3. Results

Nine PD patients were included (Table 1). Mean age at implantation was 64.0 ± 6.4 years and mean disease duration was 14.6 ± 5.9 years. DBS was refused by four patients and contraindicated in five patients (in two patients because of multi-infarctual encephalopathy, in three patients because of age).

Mean follow-up time was 5.1 ± 2.5 years (range 2–8 years). Two patients died during the follow-up period: one of lung cancer and one of heart failure. Four patients were lost to follow-up for difficulty in reaching our center.

### 3.1. Motor Efficacy

**Figure 2:**
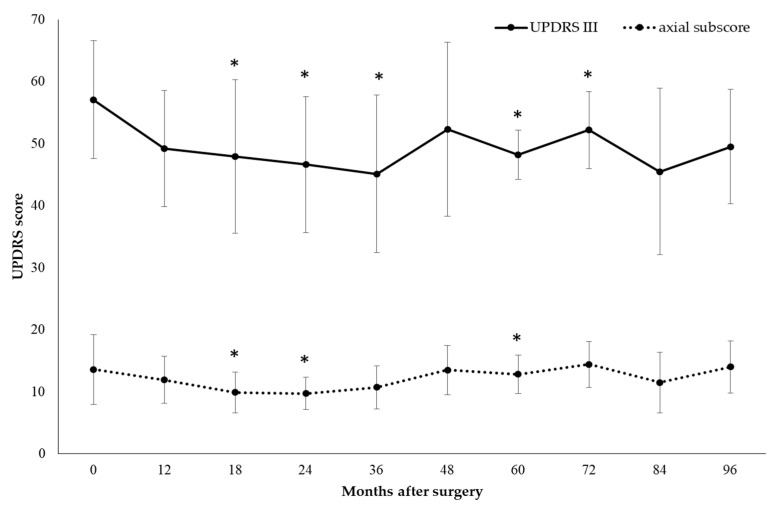
Motor efficacy of extradural motor cortex stimulation (EMCS) in the off-med condition in all patients, evaluated at different times after the implant. Error bars represent standard deviation. * *p* < 0.05 at comparisons between preoperative and postoperative scores, analyzed by means of the Wilcoxon signed-rank test.

Compared to baseline, the UPDRS-III score in the off-med condition significantly decreased by 13.8% at 12 months (*p* = 0.01), 16.1% at 18 months (*p* = 0.04), 18.4% at 24 months (*p* = 0.01), 21% at 36 months (*p* = 0.02), 15.6% at 60 months (*p* = 0.04), and 8.6% at 72 months (*p* = 0.04). Postoperative motor improvement was mostly related to decreases in the axial subscore: as compared to the baseline, in the off-med condition, the total axial subscore significantly decreased by 26.7% at 18 months (*p* = 0.02), 28.1% at 24 months (*p* = 0.03), 5.9% at 60 months (*p* = 0.04), with a slight persistent improvement up to 96 months (Figure 2).

Compared to the baseline, a progressive motor worsening was observed in the on-med condition, with a significant increase in the UPDRS-III score of 44.9% at 36 months (*p* = 0.04), 68.1% at 48 months (*p* = 0.03), 60.3% at 60 months (*p* = 0.04), 89.4% at 72 months (*p* = 0.04).

Comparisons between subgroups of patients with bilateral (*n* = 6) versus unilateral stimulation (*n* = 3) were not carried out, because of the small sample size. However, at the 24-month follow-up stage, we observed a greater improvement of the UPDRS-III score and total axial subscore in the off-med condition in bilaterally stimulated patients (18.4% decrease in UPDRS III score from 52.1 ± 9.7 at baseline to 42.5 ± 12.3 at 24 months; 29.2% decrease in axial subscore from 12.0 ± 5.7 at baseline to 8.5 ± 2.4 at 24 months), as compared to unilaterally stimulated patients (15.5% decrease in UPDRS-III score from 64.7 ± 3.1 at baseline to 54.7 ± 4.0 at 24 months; 26.9% decrease in axial subscore from 16.3 ± 2.9 at baseline to 12.0 ± 1.0 at 24 months).

**Figure 3:**
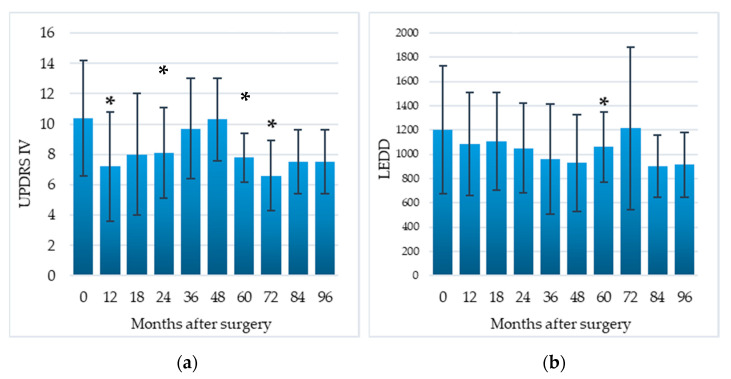
Efficacy of EMCS on motor complications (**a**) and reduction in dopaminergic therapy (**b**) in all patients, evaluated at different times after the implant. Error bars represent standard deviation. * *p* < 0.05 at comparisons between preoperative and postoperative scores, analyzed by means of the Wilcoxon signed-rank test.

Compared to the baseline, the UPDRS-IV score (assessing motor fluctuations and dyskinesias) was significantly decreased by 30.8% at 12 months (*p* = 0.03), 22.1% at 24 months (*p* = 0.02), 25% at 60 months (*p* = 0.04), 36.5% at 72 months (*p* = 0.04). Postoperatively, LEDD showed a progressive reduction, which was significant at the 60-month follow-up stage (11.8%, *p* = 0.04) (Figure 3).

All data about motor efficacy of EMCS are reported in Table 2.

### 3.2. Disease Severity

Compared to the preoperative baseline, the UPDRS total score decreased by 16.7% at 12 months (*p* = 0.01), 19.9% at 18 months (*p* = 0.02), 15.3% at 24 months (*p* = 0.03). Although not significant, the improvement was sustained up to 96 months (8.9%) (Table 2).

### 3.3. DLA and QoL

**Figure 4:**
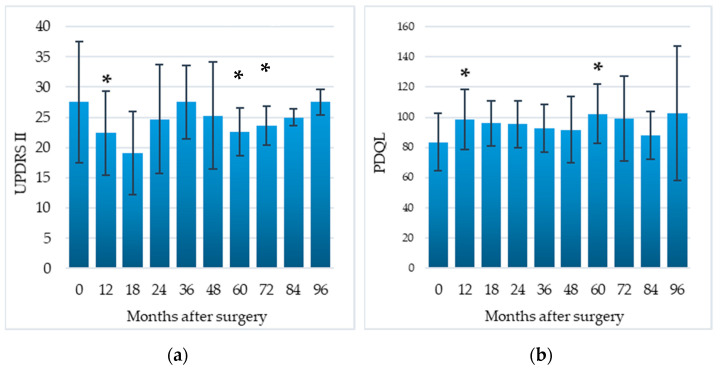
Impact of EMCS on daily living activities (**a**) and quality of life (**b**) in all patients, evaluated at different times after the implant. Error bars represent standard deviation. * *p* < 0.05 at comparisons between preoperative and postoperative scores, analyzed by means of the Wilcoxon signed-rank test.

Compared to the preoperative baseline, difficulties in DLA, assessed by the UPDRS-II, significantly decreased by 18.5% at 12 months (*p* = 0.03), 17.8% at 60 months (*p* = 0.04), 14.2% at 72 months (*p* = 0.04); QoL, assessed by means of the PDQL, significantly improved by 18.0% at 12 months (*p* = 0.02), and 22.4% at 60 months (*p* = 0.04) (Table 2, Figure 4).

### 3.4. Cognitive and Behvioral Outcome

No patients treated by EMCS developed dementia, as diagnosed according to the DSM-IV diagnostic criteria. No significant postoperative change was observed in terms of the UPDRS-I score. Postoperatively, no significant behavioral change was detected via scales assessing mood and anxiety or by clinical interviews. Improvements in MMSE (from 25.3 ± 2.6 to 26.7 ± 3.2, *p* = 0.04) and two subtests of episodic verbal memory (immediate and delayed recall of RAVLT, from 35.3 ± 6.8 to 45.9 ± 10.9 (*p* = 0.03) and from 7.0 ± 2.2 to 9.7 ± 3.0 (*p* = 0.03), respectively were detected 18 months postoperatively. Interestingly, a slight non-significant improvement was observed in the verbal phonological fluency task (from 17.3 ± 8.3 at baseline to 19.5 ± 11.9 at 12 months, 23.0 ± 13.6 at 18 months, 19.2 ± 11.0 at 36 months, 20.7 ± 9.3 at 60 months, 21.0 ± 15.6 at 96 months). No other change in cognitive performance on neuropsychological tests was detected across postoperative assessments (Table 3). Anticholinergic medications were not used in this population.

### 3.5. Safety

No serious adverse events occurred during the surgical procedure. During follow-up, no stimulation-related adverse events occurred. No patients presented seizures or epileptic discharges on postoperative electroencephalograms. In one patient, the whole implant was removed 36 months after surgery, due to an infection of the IPG pocket. Six battery replacements were performed, without complications.

## 4. Discussion

To our knowledge, the present study reports the longest clinical follow-up in PD patients treated by ECMS. This therapy induced a slight sustained motor improvement, with benefits to axial symptoms, and a reduction in motor complications and dopaminergic therapy. Despite its small size, the improvement was clinically meaningful as patients reported improvements in QoL and DLA. EMCS was safe, without detrimental effects on cognition and behavior.

In this study, we prospectively assessed the long-term efficacy and safety of EMCS in nine PD patients, followed-up for at least two years and up to eight years. EMCS showed beneficial effects on parkinsonian motor symptoms (measured by reductions in the UPDRS III in the off-medication condition) not only in the short-term, but also in the long-term follow-up. These findings, consistent with previous studies with a short-term follow-up [2,8,9,11,13], suggest that beneficial effects on motor symptoms may persist over time. Likewise, we found a progressive motor improvement up to three years after surgery. The sustained effect and progressive postoperative motor improvement may reflect processes of neural plasticity within the motor cortex, modulated by chronic stimulation [9].

Beneficial effects of EMCS were also observed on axial symptoms, which often display a poor response to levodopa and DBS [28]. This therapeutic role may be explained by the electrode position and polarity over M1. As demonstrated in 3D-volume conductor models, in bipolar EMCS, the anode of the dipole gives the largest motor response, exciting neural elements perpendicular to the electrode surface and corticofugal fibers [29]. The orientation of the electrode paddle over M1 (contact 3: 2 to 3 cm from the midline; contact 0: 4 cm more laterally) and electrode polarity (contact 3 was the anode, contact 0 was the cathode) may explain the effects on cortical areas, which are critical for axial motor function. EMCS may also modulate the activities of the cortical areas related to M1, such as the SMA, which plays a role in the pathophysiology of bradykinesia and axial symptoms [30]. Since bilateral activation of the SMA may be induced not only by bilateral but also by unilateral stimulation [6], both unilateral and bilateral EMCS stimulation may improve the axial symptoms [13,31].

As previously described [13], in our study, motor benefits were detectable only in the off-medication condition, similarly to what is seen in DBS patients. This finding rules out a synergic effect of EMCS and anti-parkinsonian medication and suggests that EMCS is able to induce motor improvements. In the on-medication condition, a progressive worsening of the UPDRS motor score was observed over time which is consistent with disease progression [32]. The absence of motor benefits detected in the on-medication condition may be explained by the remarkable effects of levodopa administration on parkinsonian motor symptoms, which might mask the slight beneficial effects of EMCS on such symptoms. Importantly, since the levodopa dose administered at each evaluation was higher than the usual morning levodopa dose, the UPDRS-III score at each evaluation might not reflect the usual on-medication condition.

In this study, EMCS was also effective in the management of PD motor complications (motor fluctuations and dyskinesias), as shown by a significant reduction in the postoperative UPDRS-IV score up to 72 months. The mean daily dose of dopaminergic drugs (measured by LEDD) showed a slight persistent decrease over time, which became significant postoperatively after 60 months. The reduction in motor complications might be explained by the slight postoperative decrease in LEDD and by a direct effect of EMCS on cortical plasticity, as suggested by transcranial magnetic stimulation studies [33,34]. Indeed, prospective EMCS studies suggested a direct effect of EMCS on dyskinesias [9,10,11]. In recent studies, the same electrodes have been used on M1 to detect cortical signs that are useful to close the loop for adaptive DBS, as a cortical narrowband gamma oscillation related to dyskinesias [35]. A direct effect of EMCS on dyskinesias may be postulated by the desynchronization of cortical narrowband gamma oscillations between the basal ganglia and cortical neurons.

EMCS also resulted in a sustained improvement of disease severity, measured by the total UPDRS score. The reduction in this score was significant up to 24 months, but despite the underlying disease progression, persisted up to 96 months of follow-up.

Not surprisingly, such beneficial effects of EMCS resulted in significant postoperative improvement of both activities of DLA (UPDRS-II score decrease) and QoL (PDQL score increase), which persisted in the long-term follow-up.

We found no detrimental effects on cognition or behavior over the long-term follow-up. We observed a significant improvement in MMSE and two subtests of episodic verbal memory only at the 18-month follow-up, which may be at least partially explained by a practice effect, and a slight (although not significant) improvement in the phonological fluency task over time, up to eight years after surgery. This latter finding is consistent with a previous observation of improvements in the fluency task in nine PD patients treated by EMCS with a 12-month follow-up. In particular, in two patients with unilateral stimulation of the left cerebral hemisphere, who showed an improvement in such fluency task at 3- and 12-month postoperative assessments [13]. In contrast, phonological fluency is consistently impaired after DBS of the subthalamic nucleus [36]. This interesting finding and other effects of EMCS on cognition should be assessed in future studies. Since EMCS induced beneficial effects on language in cases of pure akinesia with gait freezing [31] and improvements in tasks of episodic verbal memory and working memory in two patients with PD [37], it is possible that EMCS might induce beneficial effects on various cognitive processes across distinct disorders.

This study also confirms that EMCS is safe in the long-term follow-up, since adverse events occur very rarely [15]. The only adverse event in our sample was an infection of the IPG pocket, which required the removal of the whole implant three years postoperatively.

### Limitations

This study has several limitations. The first is the open-label study design, which does not allow us to rule out a placebo effect, which is nevertheless unlikely given the persistence of beneficial effects over the long-term follow-up. In fact, while it is possible that a placebo effect would occur in the first few months after the intervention, we found a sustained motor effect of EMCS beyond five years after the intervention. Moreover, this was also the case in patients with neurodegenerative disease, which usually leads to motor aggravation over the years. Indeed, beneficial effects on specific motor tasks (such as capability of rising from a chair), that did not occur immediately, but rather several months after the stimulation being switched on, reduce any potential biases deriving from the expectations of patients and physicians. Other limitations include the small number of enrolled patients and the lack of a control group. Few patients were enrolled because this treatment is intended for a highly selected population of PD patients who are not eligible for other advanced therapies. As most of the significant *p* values were marginally lower than 0.05, due to the small number of cases, they should be interpreted with caution, considering the risk of a type I error deriving from multiple comparisons. Furthermore, the availability of other effective treatments for the motor complications of PD, such as DBS or levodopa/carbidopa intestinal gel, limits the possibility to obtain a control group for standard medical treatments. Finally, the high percentage of patients lost at follow-up is an inevitable limit of long-term studies with advanced PD patients, as reported in many DBS studies. In fact, the high degree of motor disability, which characterizes this stage of disease, often leads to difficulties in reaching the center, institutionalization, and death.

## 5. Conclusions

EMCS may be a useful treatment option for advanced PD patients to reduce motor complications and dopaminergic therapy. It provides a small but sustained effect on motor symptoms (both appendicular and axial symptoms), with improvements in DLA and QoL.

Although EMCS seems to be less effective on motor symptoms than other advanced therapies (DBS or levodopa/carbidopa intestinal gel), it may be a safe alternative when these options are contraindicated or refused by patients.

Prospective controlled studies in larger samples of PD patients, possibly with prominent axial symptoms, evaluated by specific scales for axial motor symptoms and dyskinesias, are needed to further define clinical indications for EMCS in PD.

## Figures and Tables

**Table 1:** Patient’s demographic and clinical data at baseline.

Patient	Gender	Age at Surgery	UPDRS III Off-Med	UPDRS III On-Med	Hoehn and Yahr Stage	UPDRS IV	LEDD (mg)	Reason DBS Not Performed
1	F	57	8	55	24	5	13	Patient refusal
2	M	55	16	68	19	5	13	Brain atrophy
3	M	62	9	62	18	4	11	Patient refusal
4	F	57	14	64	24	5	15	Brain atrophy
5	F	66	28	61	35	5	12	Patient refusal
6	F	69	17	59	15	5	12	Age and comorbidities
7	F	70	14	62	17	5	9	Age and comorbidities
8	M	72	11	43	16	3	6	Age and comorbidities
9	M	68	14	40	18	3	3	Patient refusal
Total	4 M/5 F	64.0 ± 6.4	14.6 ± 5.9	57.1 ± 9.5	20.7 ± 6.2	4.4 ± 0.9	10.4 ± 3.8	

Abbreviations: UPDRS = Unified Parkinson’s Disease Rating Scale; DBS = deep brain stimulation.

**Table 2:** Efficacy of stimulation in all patients, evaluated at different times after ECMS implant.

	Baseline (*n* = 9)	12 m (*n* = 9)	18 m (*n* = 9)	24 m (*n* = 9)	36 m (*n* = 7)	48 m (*n* = 6)	60 m (*n* = 5)	72 m (*n* = 5)	84 m (*n* = 2)	96 m (*n* = 2)
UPDRS III med-off	57.1 ± 9.5	49.2 ± 9.4 *	47.9 ± 12.4 *	46.6 ± 11.0 *	45.1 ± 12.7 *	52.3 ± 14.0	48.2 ± 4.0 *	52.2 ± 6.2 *	45.5 ± 13.4	49.5 ± 9.2
UPDRS III axial score med-off	13.6 ± 5.6	11.9 ± 3.8	9.9 ± 3.3 *	9.7 ± 2.6 *	10.7 ± 3.5	13.5 ± 4.0	12.8 ± 3.1 *	14.4 ± 3.7	11.5 ± 4.9	14.0 ± 4.2
UPDRS III med-on	20.7 ± 6.2	27.1 ± 8.7	26.0 ± 6.5	26.7 ± 6.2	30.0 ± 7.1 *	34.8 ± 9.9 *	33.2 ± 5.1 *	39.2 ± 9.0 *	37.5 ± 13.4	45.0 ± 9.9
UPDRS IV	10.4 ± 3.8	7.2 ± 3.6 *	8.0 ± 4.0	8.1 ± 3.0 *	9.7 ± 3.3	10.3 ± 2.7	7.8 ± 1.6 *	6.6 ± 2.3 *	7.5 ± 2.1	7.5 ± 2.1
LEDD	1203.0 ± 528.1	1085.8 ± 424.5	1108.0 ± 402.3	1050.8 ± 369.3	962.4 ± 455.6	929.9 ± 401.0	1061.3 ± 286.5 *	1214.2 ± 670.8	905.0 ± 254.6	915.0 ± 268.7
UPDRS II	27.5 ± 10.0	22.4 ± 6.9 *	19.1 ± 6.9	24.7 ± 9.0	27.5 ± 6.1	25.3 ± 8.8	22.6 ± 4.0 *	23.6 ± 3.2 *	25.0 ± 1.4	27.5 ± 2.1
PDQL	83.5 ± 19.1	98.5 ± 20.0 *	96.0 ± 15.0	95.4 ± 15.5	92.7 ± 15.7	91.8 ± 22.1	102.2 ± 19.6 *	99.2 ± 28.3	88.0 ± 15.6	102.5 ± 44.5
UPDRS tot	98.8 ± 24.5	82.3 ± 16.9 *	79.1 ± 21.5 *	83.7 ± 22.1 *	89.3 ± 17.5	92.3 ± 23.8	82.8 ± 5.5	88.0 ± 8.5	83.5 ± 10.6	90.0 ± 8.5

Abbreviations: m = months; UPDRS = Unified Parkinson’s Disease Rating Scale; LEDD = levodopa equivalent daily dose; PDQL = Parkinson’s disease (PD) quality of life (QoL) questionnaire. * *p* < 0.05 at comparisons between preoperative and postoperative scores.

**Table 3:** Cognitive and behavioral evaluations in all patients, evaluated at different times after ECMS implant.

	Baseline (*n* = 9)	12 m (*n* = 9)	18 m (*n* = 9)	36 m (*n* = 7)	60 m (*n* = 5)	96 m (*n* = 2)
MMSE	25.3 ± 2.6	25.9 ± 3.0	26.7 ± 3.2 *	26.5 ± 3.9	25.0 ± 4.6	25.0 ± 2.8
Digit Span forward	4.6 ± 0.7	4.7 ± 1.0	4.4 ± 0.5	4.0 ± 0.6	5.0 ± 0	4.5 ± 0.7
Digit Span backward	3.2 ± 0.7	3.0 ± 0.7	3.0 ± 0.8	3.3 ± 0.8	3.0 ± 0	3.5 ± 0.7
Corsi’s Span forward	4.8 ± 1.3	4.6 ± 1.2	4.4 ± 1.5	4.2 ± 1.2	4.0 ± 0	3.5 ± 0.7
Corsi’s Span backward	3.8 ± 1.0	3.4 ± 1.2	3.1 ± 0.9	4.2 ± 1.5	3.7 ± 0.6	3.0 ± 0
RAVLT immediate recall	35.3 ± 6.8	42.2 ± 12.2	45.9 ± 10.9 *	32.7 ± 5.7	43.7 ± 7.0	23.5 ± 2.1
RAVLT delayed recall	7.0 ± 2.2	8.5 ± 3.7	9.7 ± 3.0 *	6.2 ± 1.2	10.0 ± 4.4	4.0 ± 0
RPM ’47	20.0 ± 7.3	21.5 ± 2.7	21.4 ± 6.9	22.7 ± 8.6	24.0 ± 9.2	19.5 ± 4.9
Phonological verbal fluency	17.3 ± 8.3	19.5 ± 11.9	23.0 ± 13.6	19.2 ± 11.0	20.7 ± 9.3	21.0 ± 15.6
Semantic verbal fluency	14.1 ± 4.0	12.7 ± 4.1	13.9 ± 3.4	14.0 ± 5.1	16.3 ± 6.8	15.0 ± 8.5
mWCST criteria	2.4 ± 1.6	2.6 ± 1.7	3.7 ± 2.1	2.8 ± 1.6	2.3 ± 2.3	1.5 ± 0.7
mWCST total errors	23.4 ± 10.4	20.5 ± 8.7	17.1 ± 11.6	19.8 ± 8.3	24.0 ± 17.6	21.0 ± 1.4
mWCST perseverative errors	8.1 ± 4.9	8.5 ± 5.6	7.0 ± 5.4	6.5 ± 3.3	6.6 ± 7.0	6.5 ± 3.5
Stroop interference time	33.7 ± 32.2	35.2 ± 17.8	35.0 ± 25.1	25.7 ± 14.0	48.3 ±55.2	37.5 ±10.6
Stroop interference errors	1.5 ± 3.9	2.2 ± 3.6	3.1 ± 4.8	2.3 ± 2.1	3.0 ± 3.3	6.0 ± 8.5
Zung Depression Scale	45.6 ± 13.6	46.4 ± 12.5	44.6 ± 8.1	47.3 ± 8.8	47.6 ± 9.3	55.0 ± 1.4
Zung Anxiety Scale	44.9 ± 12.9	47.5 ± 10.0	44.4 ± 8.0	46.8 ± 8.5	43.0 ± 11.5	44.0 ± 0

Abbreviations: m = months; MMSE = Mini-Mental State Examination; RAVLT = Rey’s Auditory Verbal Learning Test; RPM = Raven’s Progressive Matrices; mWCST = modified Wisconsin Card Sorting Test. * *p* < 0.05 at comparisons between preoperative and postoperative scores. m = months.

## Data Availability

Anonymized data will be shared with qualified external researchers, after approval of their requests.
